# The Osteoimmune Axis: Immune–Mechanical Crosstalk in Periodontal Bone Remodeling

**DOI:** 10.3390/biom16030479

**Published:** 2026-03-23

**Authors:** Anna Ewa Kuc, Grzegorz Hajduk, Paulina Kuc, Joanna Lis, Beata Kawala, Michał Sarul

**Affiliations:** 1 Department of Dentofacial Orthopedics and Orthodontics, Wroclaw Medical University, 50-425 Wroclaw, Poland; annaewakuc@wp.pl (A.E.K.); joanna.lis@umw.edu.pl (J.L.); beata.kawala@umw.edu.pl (B.K.); 2 Chair and Department of Oral Surgery, Medical University of Lublin, Witolda Chodźki 6 Street, 20-093 Lublin, Poland; 3 Faculty of Medicine, Medical University in Bialystok, ul. Kilińskiego 1, 15-089 Bialystok, Poland; 4 Department of Integrated Dentistry, Wroclaw Medical University, 50-425 Wroclaw, Poland; michal.sarul@umw.edu.pl

**Keywords:** osteoimmunology, periodontal ligament (PDL), mechanical loading, macrophage polarization (M1/M2), T-cell subsets (Th17/Treg), RANKL–osteoclast signaling, cytokine–mechanical crosstalk, oxidative stress (ROS), orthodontic bone remodeling, thin periodontal phenotype

## Abstract

**Background:** Orthodontic tooth movement is traditionally explained through mechanical deformation of the periodontal ligament (PDL); however, increasing evidence indicates that immune mechanisms critically shape bone remodeling outcomes. Mechanical stimuli influence immune cell recruitment, cytokine release, and phenotypic polarization, but these components are rarely integrated into a unified framework. **Conceptual framework:** We propose the Osteoimmune Axis Model, a conceptual framework describing how mechanical loading may bias immune polarity and thereby gate periodontal remodeling. Compressive loading appears to favor an M1 macrophage/Th17-dominant program associated with pro-inflammatory cytokines and enhanced RANKL-mediated osteoclastogenesis. In contrast, tensile or physiological strains may favor M2 macrophages and regulatory T cells (Treg), supporting IL-10, TGF-β, angiogenesis, extracellular-matrix repair, and osteoblastic activity. Stromal cells are proposed to act as mechanosensors and immune amplifiers that shape cytokine gradients and feedback loops. **Predictions:** The model predicts that identical forces may produce divergent outcomes depending on immune baseline; load duration may be more destructive than peak magnitude; tensile strain may stabilize M2/Treg pathways; thin periodontal phenotypes may shift toward the catabolic pole at lower mechanical loads; ROS may amplify immune-mediated bone loss; and immunomodulation may raise the threshold for pathological remodeling. **Conclusion:** The Osteoimmune Axis integrates mechanobiology and immunology into a testable framework for explaining variability in orthodontic periodontal remodeling and for generating hypothesis-driven, immune-aware risk assessment.

## 1. Introduction

Orthodontic tooth movement (OTM) is commonly framed as a mechanotransduction process in which compression and tension within the periodontal ligament (PDL) regulate bone resorption and formation. Yet clinically comparable mechanics can yield divergent outcomes—ranging from stable remodeling to excessive bone loss or soft-tissue breakdown—suggesting an additional biological gating layer. Accumulating evidence indicates that immune cells are mechanosensitive and that their polarization programs can dominate remodeling trajectories under load [1,2,3]. Consistent with osteoimmunology, which views skeletal and immune systems as dynamically integrated regulatory networks [4,5], we propose that orthodontic biomechanics act upstream of an immune-polarity continuum that can switch tissues between anabolic and catabolic states.

Mechanical compression induces the rapid release of inflammatory cytokines such as IL-1β, TNF-α and IL-6 from fibroblasts and resident stromal cells within the PDL [6]. These cytokines recruit innate immune cells and promote macrophage polarization toward a pro-inflammatory M1 phenotype, characterized by nitric oxide production, ROS accumulation, and stimulation of RANKL-mediated osteoclastogenesis [2,7]. Simultaneously, adaptive immune responses shift toward Th17 cell activation, further amplifying RANKL expression and inflammatory bone loss [8].

In contrast, tensile or physiologic strains generate an immunologically distinct microenvironment. Tensile loading supports M2 macrophage polarization, enhanced IL-10 signaling, regulatory T cell (Treg) induction, and improved angiogenic and anabolic responses [9,10,11]. These differences suggest the existence of a mechanically driven immune polarity, where the balance between M1/Th17 and M2/Treg dictates whether tissues undergo controlled remodeling or pathological destruction.

**Figure 1:**
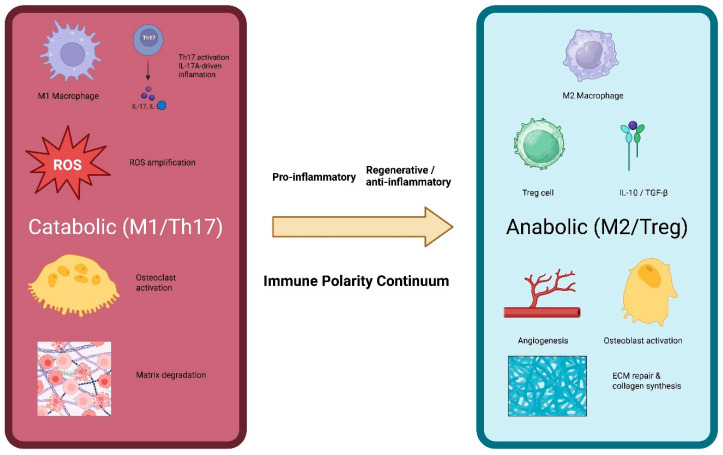
The Osteoimmune Axis Model. The Osteoimmune Axis represents a continuum of immune states ranging from catabolic (M1/Th17-dominant) to anabolic (M2/Treg-dominant) responses rather than two discrete categories. Compressive or pathological mechanical cues promote M1 macrophage and Th17 polarization, IL-17A–driven inflammation, reactive oxygen species (ROS) amplification, osteoclast activation, and extracellular matrix degradation. In contrast, tensile or physiological mechanical strain favors M2 macrophage and Treg responses, anti-inflammatory cytokine production (IL-10, TGF-β), angiogenesis, osteoblast activation, and extracellular matrix repair. This immune polarity axis links mechanical cues with tissue-specific degenerative or regenerative outcomes.

This duality forms the conceptual basis for the Osteoimmune Axis—a continuum that links mechanical stimuli to immune polarization and, ultimately, to periodontal remodeling outcomes; Figure 1. The axis helps explain why clinically similar forces may produce dramatically different results across patients, ranging from stable tooth movement to excessive bone loss or soft-tissue breakdown. It also clarifies the heightened susceptibility of the thin periodontal phenotype, which exhibits reduced vascularity, diminished ECM buffering, higher oxidative burden, and accelerated immune amplification [12,13,14].

Despite major advances in osteoimmunology and periodontal biology, these disciplines remain insufficiently integrated into orthodontic science. Existing models often isolate mechanical or inflammatory mechanisms but fail to capture the bidirectional crosstalk that governs bone remodeling under load. This perspective synthesizes current evidence to introduce the Osteoimmune Axis Model, outlining its mechanistic foundations, predicting its implications for clinical practice, and identifying opportunities for therapeutic immune modulation during orthodontic treatment. The article proposes a testable theoretical model derived from previously published mechanistic studies and does not include original experiments. While osteoimmunology and periodontal mechanobiology have been studied separately, the Osteoimmune Axis model is proposed as a unifying framework that integrates mechanical loading polarity with immune cell polarization (M1/M2 and Th17/Treg) to explain patient-specific variability in orthodontic periodontal remodeling. This hypothesis article is based on a structured narrative synthesis of previously published mechanistic, experimental, and translational studies relevant to orthodontic loading, osteoimmunology, periodontal biology, macrophage polarization, and T-cell regulation.

## 2. Background

### 2.1. Biological Basis of the Osteoimmune Axis

Orthodontic loading initiates a cascade of early molecular events within the periodontal ligament (PDL), but the immune system ultimately determines the direction and magnitude of bone remodeling. The biological foundation of the Osteoimmune Axis lies in the capacity of mechanical forces to bias immune cell polarization and, conversely, in the ability of immune pathways to amplify or restrain mechanically induced signals.

A central component of this axis is macrophage polarization. Mechanical compression and associated hypoxic or oxidative stress promote differentiation toward the M1 phenotype, characterized by elevated production of nitric oxide, TNF-α, IL-1β, and IL-6, all of which enhance osteoclast formation through RANKL-mediated pathways [15,16]. In contrast, tensile or physiological strains induce M2 macrophages, which secrete IL-10, TGF-β, and pro-angiogenic mediators that support collagen synthesis, extracellular matrix repair, and osteoblastic activity [7,17]. Thus, macrophages function as mechanosensitive immunological switches that integrate physical stimuli with tissue-level remodeling responses.

Adaptive immunity also contributes significantly to osteoimmune regulation. Pro-inflammatory Th17 cells, through secretion of IL-17A, synergize with M1 macrophages to amplify RANKL expression and osteoclastogenesis [8]. Conversely, regulatory T cells (Treg) counterbalance these effects by producing IL-10 and inhibiting excessive bone resorption [9]. Mechanical strain influences this Th17/Treg balance, shaping whether local tissues follow anabolic or catabolic trajectories.

Stromal cells—including fibroblasts, periodontal ligament stem cells and osteoblast-lineage cells—further modulate this crosstalk by acting as immune amplifiers. Under compression, they increase expression of RANKL, COX-2 and inflammatory chemokines, enhancing recruitment and polarization of immune cells [6,15]. Under tensile strain, they favor VEGF production, M2 stabilization, and collagen deposition [7].

Together, these mechanisms demonstrate that the periodontal ligament (PDL) cannot be viewed solely as a mechanical structure. Instead, it functions as an immunologically active organ whose remodeling outcomes depend on the dynamic interplay between mechanical stimuli and immune polarity. This bidirectional mechanoinflammatory integration constitutes the biological core of the Osteoimmune Axis.

### 2.2. Mechanical Loading and Immune Polarization

Mechanical forces do not only deform cells and extracellular matrices; they may also act as important regulators of immune polarity within the periodontal ligament (PDL). Compression and tension generate biomechanically distinct microenvironments that differentially shape cytokine release, metabolic state, and immune cell recruitment. These divergent conditions drive the balance between catabolic (M1/Th17) and anabolic (M2/Treg) immune responses, ultimately determining whether periodontal tissues remodel adaptively or undergo destructive resorption.

Under compressive stress, fibroblasts and stromal cells rapidly upregulate IL-1β, IL-6, TNF-α, COX-2 and CCL2, producing a cytokine milieu that promotes M1 macrophage activation [6,15,18]. M1 macrophages amplify inflammation via nitric oxide, ROS, and additional pro-inflammatory cytokines, creating a self-reinforcing loop that intensifies RANKL-dependent osteoclastogenesis [16]. Experimental models show that compression directly increases RANKL expression in PDL cells and enhances osteoclast differentiation through NF-κB and JNK pathways [16,18,19]. Simultaneously, compression skews adaptive immunity toward Th17 polarization, elevating IL-17A and downstream osteoclastic activity [8]. Th17-derived IL-17 synergizes with M1 cytokines to further potentiate bone resorption, especially under conditions of oxidative stress or pathological hypoxia. Together, these mechanisms define a mechanoinflammatory catabolic pole of the Osteoimmune Axis.

In contrast, tensile or physiological strains generate a microenvironment conducive to M2 macrophage activation, characterized by IL-10, TGF-β, arginase-1 and VEGF expression [7,17]. M2 macrophages counteract inflammatory cascades, promote endothelial stabilization, facilitate ECM repair, and encourage osteoblastic activity—hallmarks of the anabolic pole of the Osteoimmune Axis. Tensile strain also enhances Treg differentiation, increasing IL-10 and suppressing excess RANKL expression [9,20]. These anti-inflammatory pathways restore tissue homeostasis and limit destructive remodeling. Mechanically induced Treg responses represent an underappreciated regulatory layer that may explain why some patients maintain stable periodontal tissues during orthodontic treatment despite chronic loading.

In simplified terms, compression may promote a hypoxia/ROS-enriched milieu that favors M1/Th17 polarization and increased RANKL signaling, whereas tension may support perfusion, VEGF-associated responses, and M2/Treg stabilization with enhanced extracellular matrix repair. These reciprocal feedback systems suggest that immune polarity is not merely a secondary consequence of mechanical load, but part of a mechanosensitive network shaping tissue fate. The Osteoimmune Axis therefore reflects the integrated contribution of mechanical deformation, cytokine gradients, immune cell polarization, and bone remodeling dynamics.

### 2.3. Osteoimmune Determinants of Bone Remodeling

Bone remodeling during orthodontic tooth movement (OTM) is governed not simply by mechanical deformation of the periodontal ligament (PDL), but by immune–bone crosstalk that amplifies or attenuates the effects of load. Mechanical signals modulate the recruitment, phenotype and activity of immune cells, which in turn regulate osteoclast and osteoblast differentiation, coupling, and turnover. The Osteoimmune Axis conceptualizes this as a continuum in which immune polarity is the decisive factor controlling whether mechanical loading results in adaptive remodeling or pathological destruction.

Osteoclast differentiation is highly sensitive to immune status. Pro-inflammatory cytokines such as TNF-α, IL-1β, IL-6 and IL-17 enhance RANKL expression in PDL fibroblasts, osteoblast-lineage cells and T-cells, thereby accelerating osteoclastogenesis under compressive load [2,6,8]. M1 macrophages produce nitric oxide and ROS, further amplifying RANKL signaling and directly stimulating osteoclast precursors [15,16,21]. Th17-derived IL-17 synergizes with TNF-α and IL-6 to drive osteoclast formation even at sub-threshold mechanical loads, linking immune polarity to the sensitivity of bone resorption pathways [8].

The bone-forming compartment is equally influenced by immune signals. Anti-inflammatory environments dominated by M2 macrophages and Treg cells enhance osteoblast differentiation through IL-10, TGF-β and pro-angiogenic mediators [7,9,17]. M2 macrophages secrete osteogenic exosomes and support endothelial stabilization, promoting conditions that facilitate osteoblast function and mineralization [22]. Conversely, chronic M1/Th17 signaling suppresses osteoblast activity, increases oxidative stress, and impairs matrix deposition, hindering regenerative potential during OTM [21,23,24].

Bone remodeling requires coordinated vascular adaptation. VEGF-driven angiogenesis is essential for osteoclast recruitment and osteoblast precursor delivery. Mechanical tension generally promotes angiogenic stabilization, whereas compression, hypoxia and ROS impair capillary formation [10,23]. M2 macrophages function as “angiogenic supervisors,” while M1 macrophages destabilize endothelium and inhibit vascular recovery [7,22]. This vascular dimension reinforces the Osteoimmune Axis by linking immune polarity to perfusion recovery and thus to the tissue’s ability to exit inflammatory, catabolic states.

PDL fibroblasts and osteoblast-lineage stromal cells are not passive targets of inflammation. Instead, they act as immune amplifiers, responding to mechanical cues with changes in cytokine production and antigen presentation [6,18]. Under compression, stromal cells increase IL-6, CCL2 and RANKL, recruiting additional M1 macrophages and Th17 cells; under tension, they increase IL-10, VEGF and ECM synthesis, reinforcing M2/Treg stabilization [7,9,20]. These stromal–immune feedback loops determine whether bone remodeling proceeds in a balanced, controlled manner or shifts toward destructive inflammation. Together, these pathways demonstrate that bone remodeling during OTM is not simply a mechanically induced phenomenon but a mechanoinflammatory process dominated by immune polarity. The Osteoimmune Axis captures this interplay, providing a conceptual foundation for understanding how mechanical and immunological inputs jointly dictate bone turnover [25,26].

### 2.4. Thin Periodontal Phenotype and Immune Susceptibility

The thin periodontal phenotype represents a biological state of reduced vascularity, diminished extracellular matrix (ECM) buffering, and heightened inflammatory reactivity, rendering these tissues intrinsically more susceptible to osteoimmune dysregulation. This phenotype is characterized by minimal gingival thickness, reduced supracrestal soft-tissue volume, and a thin buccal cortical plate—features that diminish both mechanical and immunological resilience under orthodontic load [12,13,14].

Thin phenotypes may have reduced vascular reserve and limited perfusion adaptability, which can lower baseline oxygen availability and predispose tissues to hypoxia under even modest compressive forces; osteoimmune pathways can amplify this shift [8,9,27]. Because hypoxia and oxidative stress potentiate M1 macrophage and Th17 activation, patients with thin tissues enter the catabolic immune polarity of the Osteoimmune Axis more rapidly than those with thick phenotypes. This explains why seemingly light orthodontic forces may produce disproportionately severe inflammatory and resorptive outcomes in susceptible individuals.

The ECM in thin gingival and PDL tissues has lower viscoelastic capacity, reduced collagen density, and impaired mechanical damping. These properties increase local strain concentrations and facilitate the rapid diffusion of IL-1β, TNF-α, IL-6 and IL-17 into deeper periodontal compartments [6,15,18]. Enhanced cytokine penetration intensifies M1/Th17 polarization and amplifies downstream RANKL-mediated osteoclastogenesis. The periodontal ligament is mechanically and compositionally nonuniform, which can further concentrate local strains under sustained loading [28].

Conversely, thick phenotypes—with greater ECM density, water content, and viscoelastic resistance—buffer both mechanical strain and inflammatory mediators, maintaining immune polarity closer to the M2/Treg axis.

Thin phenotypes may enter an oxidative-stress–amplifying state (ROS) earlier under orthodontic loading, consistent with limited vascular reserve and a more pronounced pro-inflammatory milieu under compression [15,16,23,27]. ROS synergize with M1 and Th17 mediators to drive osteoclastic activity and suppress osteoblastic regeneration [21,23,24]. This creates a biochemical environment in which bone loss, collagen degradation, and impaired angiogenesis become self-reinforcing.

Clinical studies consistently demonstrate that thin periodontal phenotypes show higher incidences of recession, crestal bone loss, and dehiscence during orthodontic movement, independent of force magnitude [12,13,14,29,30]. This aligns with the Osteoimmune Axis, in which lower mechanical–immunological thresholds facilitate rapid shifts toward catabolic pathways. CBCT and histologic analyses show that reduced cortical thickness and soft-tissue volume correlate with elevated inflammatory markers, increased osteoclast density, and decreased angiogenic potential under load [29,30,31], reinforcing the interpretation of thin phenotype as an immunologically hypersensitive state.

Taken together, thin periodontal phenotypes should be understood not simply as an anatomical descriptor, but as a biological vulnerability state defined by low vascular reserve, impaired ECM buffering, high oxidative reactivity, and accelerated immune polarization. These factors collectively lower the threshold for entry into the destructive pole of the Osteoimmune Axis, thereby explaining the clinical variability observed among orthodontic patients.

### 2.5. The Osteoimmune Axis Model

The Osteoimmune Axis Model conceptualizes the periodontal response to orthodontic mechanical loading as a continuum governed by immune polarity, rather than by force magnitude alone. Mechanical inputs—compression, tension, shear and strain rate—shape cytokine landscapes, drive immune cell polarization, and determine whether bone remodeling proceeds along anabolic (M2/Treg) or catabolic (M1/Th17) pathways. This framework integrates mechanotransduction, immune signaling and stromal biology into a unified explanatory model of periodontal tissue fate.

Mechanical compression initiates a well-characterized cascade involving hypoxia, ROS generation and inflammatory cytokine release from fibroblasts and stromal cells [6,15]. These signals recruit monocytes and polarize them toward the M1 macrophage phenotype, marked by high TNF-α, IL-1β, NO and ROS production [18,21]. M1 macrophages in turn enhance RANKL expression and sensitize osteoclast precursors, strongly promoting bone resorption [16]. In parallel, compression shifts adaptive immunity toward Th17 polarization, increasing IL-17A release and synergizing with TNF-α and IL-6 to amplify osteoclastic activity [8,24]. Th17 cells also promote endothelial destabilization and impair angiogenic recovery, further prolonging the inflammatory environment [32]. Together, these processes create a self-reinforcing catabolic loop, in which mechanical compression and immune activation amplify one another.

Tensile or physiologic strain produces a microenvironment that counterbalances destructive remodeling. Tensile loading increases perfusion, enhances VEGF expression and decreases ROS accumulation, creating conditions that favor M2 macrophage polarization [7,17]. M2 macrophages secrete IL-10, TGF-β and pro-angiogenic mediators, promote collagen synthesis and stabilize the vasculature, supporting osteoblast survival and differentiation [22,23]. Simultaneously, tensile strain induces Treg differentiation, elevating IL-10 and suppressing excessive RANKL expression [9]. Treg cells counterbalance Th17-mediated bone destruction and restore homeostasis within the PDL. The combined effects position M2/Treg immunity as the anabolic pole of the Osteoimmune Axis—where remodeling is regulated, balanced and capable of supporting regeneration.

A defining feature of the Osteoimmune Axis is its threshold-dependent switching between anabolic and catabolic states. Small shifts in mechanical load can dramatically alter immune polarity once critical thresholds of cytokines, ROS or vascular collapse are reached. This aligns with experimental findings in osteoimmunology showing non-linear transitions in bone remodeling behavior under inflammatory versus regenerative control [25,26,33,34,35,36,37].

Once the catabolic threshold is crossed, positive-feedback loops—hypoxia → ROS → M1 → Th17 → RANKL → osteoclasts—drive rapid escalation of bone resorption [4,8,21,24]. Conversely, conditions favoring perfusion and anti-inflammatory cytokines reinforce M2/Treg stability and promote efficient bone formation [7,9,17]. Thus, periodontal response is not a simple function of force, but of mechanically induced immune polarity, modulated by tissue phenotype, vascular reserve, oxidative buffering capacity and inflammatory baseline.

In this model, “threshold-dependent switching” does not imply a single universal numeric cut-off; rather, it describes a state transition that occurs when a coordinated set of signals crosses a critical range—typically involving pro-inflammatory cytokine escalation, oxidative stress amplification, and/or microvascular compromise. Practically, threshold behavior can be operationalized by tracking a composite immune-polarity state (catabolic M1/Th17–weighted signals vs anabolic M2/Treg–weighted signals) together with perfusion/oxygenation and ROS-related measures. A shift toward the catabolic pole is inferred when pro-inflammatory/osteoclastogenic mediators rise while perfusion falls and oxidative stress increases—consistent with the model’s positive-feedback cascade (hypoxia → ROS → M1/Th17 → RANKL → osteoclastogenesis)—Table 1.

Together, these readouts could be combined into a pragmatic Immune Polarity Index, conceptually defined as the balance between catabolic (M1/Th17-associated) and anabolic (M2/Treg-associated) signaling within periodontal tissues. Rather than a fixed clinical score at present, this index should be understood as a testable composite construct that future studies may operationalize using cytokine ratios, oxidative stress markers, and immune cell composition in gingival crevicular fluid or periodontal tissues.

Importantly, the transition between anabolic and catabolic remodeling states should not be interpreted as a binary process. Instead, available evidence suggests the existence of a biological “therapeutic window” in which mechanical loading can be accommodated without triggering destructive inflammatory amplification. When mechanical stress exceeds the adaptive capacity of the periodontal extracellular matrix, immune signaling may shift toward catabolic pathways characterized by ROS accumulation, pro-inflammatory cytokine release, and enhanced osteoclastogenesis. Conversely, within the adaptive window, reparative pathways dominated by M2 macrophages and regulatory T cells may prevail, supporting tissue remodeling and homeostasis. The Osteoimmune Axis therefore conceptualizes periodontal response as a dynamic balance between these opposing states rather than a fixed biological outcome.

The Osteoimmune Axis interfaces directly with mechanical polarity and hypoxia-based frameworks. Compressive polarity accelerates M1/Th17 activation via hypoxia and ROS, while tensile polarity stabilizes M2/Treg responses through improved perfusion and angiogenesis [9,18,19]. The thin periodontal phenotype—featuring lower oxygen reserve and reduced ECM buffering—crosses destructive thresholds at much lower loads [12,13,14,30,31]. This multiscale integration provides a biologically plausible explanation for clinical variability and highlights immune polarity as the master regulator translating mechanical signals into periodontal tissue outcomes.

## 3. Discussion

### 3.1. Predictions of the Osteoimmune Axis

The Osteoimmune Axis Model generates several testable, mechanistically grounded predictions that extend beyond traditional orthodontic biomechanics and provide a biologically coherent explanation for interindividual variability in tissue response.

Prediction 1—Identical mechanical forces will produce different outcomes depending on immune baseline.

Patients with elevated inflammatory tone (e.g., high IL-6, IL-1β, TNF-α), periodontal inflammation, or systemic immune priming will display lower thresholds for transitioning into the M1/Th17-dominant catabolic pole. Experimental work in osteoimmunology shows that inflammatory priming dramatically accelerates osteoclastogenesis and bone loss under mechanical or metabolic challenge [21,38].

Therefore, two patients receiving identical orthodontic forces may experience divergent outcomes—ranging from controlled remodeling to rapid bone resorption—depending on immune status.

Prediction 2—Persistence of catabolic immune polarity predicts breakdown better than peak force.

The model predicts that the duration of M1/Th17-dominant occupancy (and delayed resolution toward M2/Treg) is a stronger predictor of pathological resorption than the instantaneous force peak. Intermittent mechanics that allow immune resolution should shorten the catabolic “time-in-state” and shift the response toward regulated remodeling. [16,18,19].

Prediction 3—Tensile loading promotes an M2/Treg-protective phenotype that can counterbalance compressive inflammation.

Even in mixed loading fields, regions under tension should exhibit enhanced IL-10, TGF-β, VEGF and M2 stabilization, reducing inflammatory overshoot in adjacent compressed areas. Work in stromal mechanobiology shows that tensile strain recruits M2 macrophages and suppresses pro-inflammatory cytokine expression [7,9,20].

Prediction 4—Thin periodontal phenotype will enter the destructive immune pole at lower loads.

Because thin phenotypes have reduced vascular reserve, increased oxidative stress, lower ECM buffering, and higher cytokine diffusivity, they should reach M1/Th17 dominance significantly earlier than thick phenotypes [12,13,14,27,28,29,30,31]. This explains clinical observations of greater recession and dehiscence risk in thin periodontal tissues even under “light” forces [14,29,30].

Prediction 5—ROS amplifies immune polarity switching.

The model predicts that elevated ROS synergizes with inflammatory cytokines to bias macrophage and T-cell polarization toward M1/Th17 and to stabilize osteoclastogenic coupling, effectively lowering the immune-polarity threshold for pathological remodeling [21,22,23,39,40].

Prediction 6—Immune-modulating strategies can raise the mechanical safety threshold.

If immune polarity governs remodeling fate, then interventions that reduce M1/Th17 signaling and/or promote M2/Treg stabilization (e.g., targeting IL-17/TNF pathways, enhancing regulatory signaling, macrophage-polarizing or pro-resolving approaches) should increase the threshold for pathological resorption under load [24,35,41,42].

### 3.2. Clinical Implications

The Osteoimmune Axis reframes orthodontic biology by highlighting immune polarity as a key modulator of how force magnitude, duration, and distribution translate into periodontal remodeling outcomes. This perspective has several clinically meaningful implications.

Individuals with elevated inflammatory burden (e.g., gingivitis, periodontitis, metabolic inflammation, systemic cytokine elevation) are predicted to enter the M1/Th17-dominant state earlier, lowering the threshold for bone resorption and soft-tissue breakdown [21,38]. This suggests that pre-treatment immune profiling or inflammation control may significantly reduce periodontal risk.

Thin phenotypes, characterized by low vascular reserve and limited ECM buffering, exhibit rapid entry into catabolic immune polarity under load [12,13,14,27,28,29,30,31]. These patients may benefit from: reduced sustained compression, staged biomechanics, early soft-tissue augmentation, and increased monitoring of inflammatory responses. Sustained compression leads to persistent M1/Th17 activation, whereas intermittent or physiologic strains promote M2/Treg stabilization [18,19,20]. Thus, force duration and distribution are more critical predictors of tissue breakdown than the absolute force value.

Mechanistically targeted interventions—such as antioxidants, IL-17 inhibitors, TNF-α modulators, or M2-polarizing agents—have the potential to shift tissues away from catabolic immune polarity and enhance periodontal safety [24,39].

This model provides a compelling explanation for why identical orthodontic mechanics yield divergent outcomes across patients: the immune system acts as the amplifier of mechanical signals, determining whether tissues remodel adaptively or destructively.

From a clinical perspective, the Osteoimmune Axis model suggests that orthodontic outcomes may depend not only on mechanical parameters such as force magnitude or direction, but also on the baseline immune status of periodontal tissues. This concept introduces the possibility of integrating immunological risk assessment into orthodontic treatment planning. Patients presenting with elevated inflammatory markers or oxidative stress indicators may exhibit a higher probability of catabolic remodeling responses during orthodontic loading.

In practical terms, future clinical implementation of the Osteoimmune Axis could involve chairside or laboratory evaluation of biomarkers derived from gingival crevicular fluid (GCF). Cytokines such as IL-6, IL-17, TNF-α, and RANKL, as well as oxidative stress markers, may provide insight into the patient’s baseline immune polarity. Such information could potentially guide individualized force application strategies, treatment timing, or adjunctive anti-inflammatory interventions aimed at reducing pathological remodeling.

Furthermore, the model supports the concept of phenotype-based orthodontics. Patients with thin periodontal phenotypes or pre-existing inflammatory susceptibility may require modified biomechanical protocols, including lower force magnitudes, extended activation intervals, or enhanced periodontal monitoring. By integrating mechanical and immunological factors, the Osteoimmune Axis may therefore contribute to the development of personalized orthodontic strategies aimed at improving tissue stability and reducing adverse remodeling.

### 3.3. Future Directions

The Osteoimmune Axis creates new opportunities for translational research and clinical innovation. Salivary or crevicular biomarkers reflecting IL-6, IL-17, TNF-α, IL-10, oxidative stress markers, or macrophage-derived mediators could help identify patients at risk of immune-driven bone loss under orthodontic load [43]. Emerging modalities such as laser Doppler flowmetry, OCT angiography, and near-infrared spectroscopy may allow for the visualization of perfusion and inflammatory gradients in the PDL during force application [44]. Mapping these gradients could validate threshold behavior predicted by the model. Drugs that shift macrophage or T-cell polarity (e.g., IL-17 blockade, IL-10 agonists, NF-κB inhibitors, resolvins) may raise the threshold for destructive remodeling. Preclinical studies demonstrate dramatic reductions in bone loss when immune pathways are modulated under mechanical challenge [19,24]. Multiscale models combining mechanical strain, cytokine diffusion, immune cell kinetics, and osteoclast/osteoblast dynamics could quantitatively predict axis transitions and patient-specific risks [45]. These models would enable immune-aware orthodontic planning. Future clinical trials should investigate tailored orthodontic strategies for thin phenotypes, incorporating shorter compression durations, controlled strain trajectories, and immune-modulating adjuncts to prevent pathological remodeling [12,13,14,29,30,31]. Because immune polarity and perfusion dynamics shape bone turnover and regenerative outcomes across inflammatory contexts, this model may unify observations across skeletal biology and guide regenerative strategies [27,46].

### 3.4. Limitations

This article presents a conceptual and hypothesis-driven framework rather than a direct experimental demonstration of immune–mechanical causality in orthodontic periodontal remodeling. Several relationships discussed in the model are supported by indirect or translational evidence derived from osteoimmunology, periodontal biology, and mechanobiology, but not all have been validated specifically in orthodontic human tissues. In addition, the proposed Immune Polarity Index remains a conceptual construct that requires prospective validation and operational definition. The model should therefore be interpreted as a biologically grounded framework intended to generate testable hypotheses rather than as a definitive explanatory system.

## 4. Conclusions

The Osteoimmune Axis Model proposes that periodontal responses to orthodontic loading are shaped not only by biomechanics, but also by immune polarity along an M1/Th17-to-M2/Treg continuum. By integrating mechanotransduction, inflammatory signaling, oxidative stress, and tissue phenotype, the model offers a testable explanation for patient-specific variability in periodontal remodeling. This framework may support future development of immune-aware orthodontic risk assessment, phenotype-based treatment planning, and targeted strategies aimed at reducing pathological remodeling during tooth movement.

## Figures and Tables

**Table 1:** Candidate readouts to test threshold-like switching in the Osteoimmune Axis.

Model Domain	Candidate Readout (Examples)	Sample/Modality	Interpretation in the Osteoimmune Axis
Perfusion/oxygenation (“vascular reserve”)	Relative perfusion, microvascular density proxies; oxygenation surrogates	Chairside/Imaging where available (e.g., laser Doppler, OCT angiography, near-infrared methods)	Declining perfusion/oxygenation supports proximity to a catabolic switch, consistent with “vascular collapse” as a threshold trigger.
Oxidative stress (ROS burden)	Oxidative stress markers; antioxidant capacity surrogates	GCF/saliva (research setting)	Rising ROS burden supports a catabolic shift and self-amplification of inflammation.
Catabolic cytokine load (M1/Th17 pole)	IL-1β, TNF-α, IL-6; IL-17A as Th17 axis readout	GCF/saliva; tissue-level assays (research)	Increasing catabolic cytokines indicates drift toward the M1/Th17 pole that promotes osteoclastogenesis.
Anabolic/regulatory signaling (M2/Treg pole)	IL-10, TGF-β (regulatory tone)	GCF/saliva; tissue-level assays (research)	Higher regulatory signals indicate stabilization of the M2/Treg pole and buffering against catabolic switching.
Osteoclastogenic coupling	RANKL-related readouts (e.g., RANKL/OPG conceptual ratio), osteoclast activity surrogates	GCF/tissue-level assays (research)	Rising osteoclastogenic signaling supports that the axis has entered (or is approaching) the destructive catabolic state.
Immune polarity (cellular composition)	M1/M2 balance; Th17/Treg balance (marker-based)	Tissue immunostaining/flow (research)	A quantitative “immune polarity index” can summarize whether the tissue state is catabolic (M1/Th17) vs anabolic (M2/Treg).

## Data Availability

No new data were generated or analyzed in this study.
